# LGF‐Net: A multi‐scale feature fusion network for thyroid nodule ultrasound image classification

**DOI:** 10.1002/acm2.70149

**Published:** 2025-07-25

**Authors:** Yao Xiao, Yan Zhuang, Wenwu Ling, Shouyu Jiang, Ke Chen, Guoliang Liao, Yuhua Xie, Yao Hou, Lin Han, Zhan Hua, Yan Luo, Jiangli Lin

**Affiliations:** ^1^ College of Biomedical Engineering Sichuan University Chengdu China; ^2^ Department of Ultrasound West China Hospital Sichuan University Chengdu China; ^3^ Department of General Surgery China‐Japan Friendship Hospital Beijing China

**Keywords:** feature fusion, hybrid network, thyroid nodules, ultrasound image

## Abstract

**Background:**

Thyroid cancer is one of the most common cancers in clinical practice, and accurate classification of thyroid nodule ultrasound images is crucial for computer‐aided diagnosis. Models based on a convolutional neural network (CNN) or a transformer struggle to integrate local and global features, which impacts the recognition accuracy.

**Purpose:**

Our method is designed to capture both the key local fine‐grained features and the global spatial features essential for thyroid nodule diagnosis simultaneously. It adapts to the irregular morphology of thyroid nodules, dynamically focuses on the key pixel‐level regions of thyroid nodules, and thereby improves the model's recognition accuracy and generalization ability.

**Methods:**

The proposed multi‐scale fusion model, the local and global feature fusion network (LGF‐Net), inspired by the dual‐path mechanism of human visual diagnosis, consists of two branches: a CNN branch and a Transformer branch. The CNN branch integrates the wavelet transform and deformable convolution module (WTDCM) to enhance the model's ability to capture discriminative local features and recognize fine‐grained textures. By introducing the aggregated attention (AA) mechanism, which mimics biological vision, into the Transformer branch, spatial features are effectively captured. The adaptive feature fusion module (FFM) is then utilized to integrate the multi‐scale features of thyroid nodules, further improving classification performance.

**Results:**

We evaluated our model on the public thyroid nodule classification dataset (TNCD) and a private clinical dataset using accuracy, recall, precision, and F1‐score. On TNCD, the model achieved 81.50%, 79.51%, 79.92%, and 79.70%, respectively. On the private dataset, it reached 91.24%, 88.90%, 90.73%, and 89.73%, respectively. These results outperformed state‐of‐the‐art methods. We also conducted ablation studies and visualization analysis to validate the model's components and interpretability.

**Conclusions:**

The experiments demonstrate that our method improves the accuracy of thyroid nodule recognition, shows its strong generalization ability and potential for clinical application, and provides interpretability for clinicians' diagnoses.

## 1 INTRODUCTION

Thyroid nodules are among the most common endocrine cancers and nodular lesions. According to the 2024 statistics from the International Agency for Research on Cancer, thyroid cancer ranks among the top ten cancers in terms of both incidence and mortality,^1^, ^2^ posing a significant public health threat.

**FIGURE 1:**
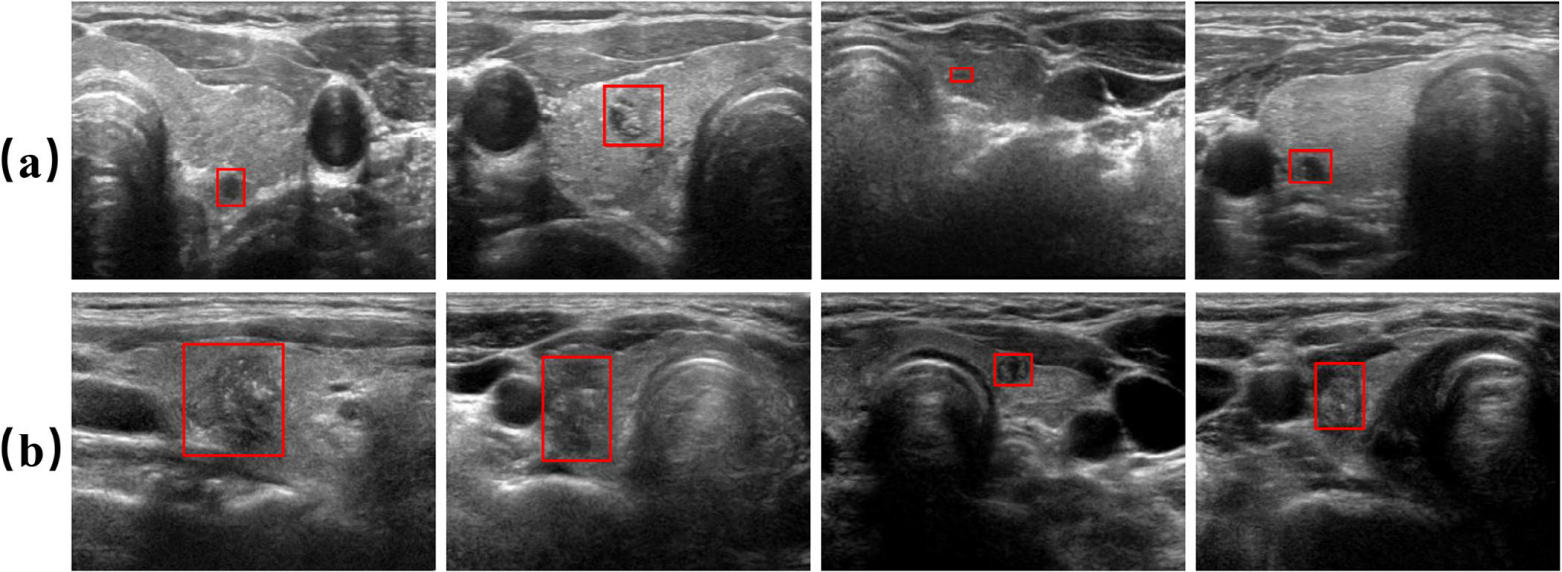
Examples of thyroid nodules in ultrasound images. (a) benign, (b) malignant. The red box marks the location and shape of the nodule.

In clinical practice, radiologists assess features such as composition, echogenicity, margins, and shape of nodules in thyroid ultrasound images to assign a grade using the thyroid imaging reporting and data system (TI‐RADS).^3^ However, due to the marked differences in the characteristics of thyroid nodules across different risk levels and the complex morphological variations observed in these nodules (as shown in Figure 1), the process of grading nodules predominantly depends on the clinical expertise and technical skills possessed by radiologists. The TI‐RADS grading results are susceptible to subjective judgment, leading to a risk of missed malignant cases. Thus, accurately and automatically classifying thyroid nodules from ultrasound images remains a challenging task.

With the advancement of deep learning, methods based on convolutional neural networks (CNNs) have been extensively used in medical image recognition tasks.^4^, ^5^ Due to CNNs’ ability to automatically capture image features, numerous CNN‐based algorithms for thyroid nodule diagnosis have been proposed.^6^, ^7^, ^8^, ^9^ However, CNNs are limited by the size of the convolution kernels and tend to focus more on learning local features. The downsampling and pooling operations in CNNs can lead to the loss of contextual and spatial information, such as the thyroid nodule's position, surrounding echogenicity, and relative size—critical attributes for clinical diagnosis—thereby affecting classification performance. The transformer,^10^ initially designed for global modeling in natural language processing, has shown remarkable effectiveness in managing long‐range dependencies. The Vision Transformer (ViT)^11^ was the pioneering model that introduced the Transformer architecture to computer vision, particularly for image recognition tasks. Since then, numerous Transformer‐based algorithms for thyroid recognition^12^, ^13^, ^14^, ^15^ have been proposed. By dividing ultrasound images into multiple patches and inputting these patches as a sequence, the Transformer treats each patch's interaction with all other patches equally due to its self‐attention mechanism. However, this global interaction may hinder the model's ability to capture local neighborhood features as effectively as CNNs. In thyroid nodule classification, important information, such as nodule echogenicity, texture, margins, and aspect ratio, may be overlooked, making recognition more difficult.

CNNs can automatically extract local features and outperform transformers in computational efficiency and data volume, but they have a weaker global perception of critical anatomical structures. Transformers excel at handling spatial information and can effectively learn image features, but they are less efficient at extracting local features and require substantial computational resources and large datasets. Furthermore, inherent features of thyroid ultrasound images, such as the high complexity of nodule positions and significant differences in nodule shape and size, add to the difficulty of model training.

To address these issues, we introduce a multi‐scale fusion method that integrates CNN and Transformer to effectively utilize features such as texture, color, and spatial information from ultrasound images for thyroid nodule diagnosis.

Thus, the main contributions of this study are as follows:

1. We introduced a dual‐branch parallel network architecture called the local and global feature fusion network (LGF‐Net), which combines CNN and transformer to extract local and global features simultaneously.
2. We implemented a feature fusion module (FFM) that adapts to effectively merge features from CNN and transformer branches at multiple scales. By leveraging channel and spatial attention (SA) mechanisms and utilizing shortcut connections to integrate features, the system effectively prioritizes important feature channels and regions.
3. To address the complex geometric deformations of thyroid nodules in ultrasound images, we designed a novel method in the process of local feature extraction, the wavelet transform and deformable convolution module (WTDCM). By introducing adaptive offsets, WTDCM enhances the model's ability to accommodate irregular nodule shapes while accurately capturing multi‐scale features.
4. We introduced the aggregated attention (AA) mechanism in global feature extraction to simulate the dynamic focus mechanism of biological vision, enabling precise localization of key thyroid nodule regions. This improves feature extraction accuracy and enhances the model's discriminative capability.
5. We evaluated our automated nodule diagnosis model using a multi‐center thyroid nodule ultrasound dataset. The model demonstrated strong diagnostic performance and cross‐domain generalization ability.

## 2 RELATED WORKS

### 2.1 CNN‐based methods

In recent years, driven by the rapid advancement of deep learning techniques, CNNs have played a significant role in disease screening tasks.^5^, ^16^, ^17^ CNNs can automatically learn complex features from images without the need for manual feature engineering, which reduces reliance on specialized knowledge and improves efficiency. In the task of thyroid ultrasound image classification, CNNs have demonstrated classification accuracy comparable to, or even surpassing, that of expert physicians. For example, Wang et al.^18^ compared the performance of the VGG16 deep learning model with traditional radiomics‐based machine learning methods in classifying thyroid nodules from ultrasound images, demonstrating the superiority of deep learning approaches for this task. Wang et al.^19^ developed an attention‐based multi‐channel convolutional model to differentiate between benign and malignant thyroid nodules. Deng et al.^20^ designed a multitask branching attention CNN network to perform risk stratification of nodules based on TI‐RADS, enhancing the feature differentiation between benign and malignant nodules. To help the model focus on the nodule region, Lu et al.^21^ and Tang et al.^22^ proposed an online class activation mapping (CAM) mechanism^23^ into CNNs to capture finer features of benign and malignant nodules. However, CNNs suffer from the loss of global features during training, which can significantly impact thyroid nodule classification. To address this, Wang et al.^8^ designed a network with hybrid convolutional and skip blocks to expand the receptive field and capture more spatial information from images. Li et al.^24^ introduced a deformable SA mechanism to extend the convolutional sampling positions. Zhao et al.^25^ introduced a deep neural network (DNN)‐based feature disentangling method specifically for thyroid ultrasound images to learn representations of both local and global features.

Although CNN‐based methods are effective at extracting local features, such as texture and color, and can capture some contextual information through convolution improvements, they still face limitations in capturing dependencies across the image due to the constraints of convolutional kernel sizes.

### 2.2 Transformer‐based methods

With the introduction of Transformer models, an increasing number of researchers have explored their application in medical image processing, with studies indicating that transformers have outperformed CNNs in many computer vision tasks.^26^, ^27^ Ma et al.^12^ benchmarked various supervised and self‐supervised pre‐training methods in medical classification tasks using different transformer variants. Ding et al.^28^ proposed a multi‐view breast ultrasound feature extraction method based on an improved Swin Transformer. By introducing a stereo attention module, the model significantly enhances lesion localization accuracy and achieves efficient classification of benign and malignant breast nodules. Sun et al.^13^ proposed a Vision‐Transformer‐based model for thyroid nodule classification, employing contrastive learning to improve diagnostic accuracy and biopsy recommendation specificity. Baima et al.^14^ introduced the Dense Nodal Swin‐Transformer method for diagnosing thyroid nodules, which segments images into patches and leverages multi‐layer features to enhance diagnostic performance. To enhance model interpretability, Wang et al.^15^ introduced a dual‐branch network featuring an attention‐based integration module. This module combines region‐based and boundary‐based domain knowledge from TI‐RADS and employs a progressive training approach to efficiently learn domain‐specific features, leading to consistent improvements in the performance of benign‐malignant classification tasks.

However, Transformer‐based methods require a large dataset and lack sensitivity to local features. As a result, these methods still face challenges in focusing on the critical details of thyroid nodules.

### 2.3 CNN and transformer hybrid methods

In thyroid nodule classification, models not only need to identify the nodule region but also focus on the entire anatomical structure where the nodule is located. To address this, researchers have designed CNN‐Transformer hybrid models for medical image tasks,^29^, ^30^ combining CNNs' local feature extraction capability with Transformers' ability to capture contextual information. Huang et al.^31^ incorporated residual blocks into the Swin Transformer, enabling the model to better adapt to the characteristics of thyroid ultrasound images and enhancing its sensitivity to both global and local features of thyroid nodules. Zhang et al.^32^ fused ultrasound and infrared thermal imaging features that are extracted by CNN and Transformer through different encoding modules. Lu et al.^33^ combined convolutional feature maps and self‐attention maps through exponential weighting. Chen et al.^34^ designed an attribute enhancement module to capture the fine‐grained and coarse‐grained features of images, thereby improving thyroid nodule classification accuracy.

Although CNN‐Transformer hybrid models have been widely applied to thyroid nodule classification and have improved performance to some extent, challenges remain in effectively integrating global and local features and accurately focusing on key nodule regions. On one hand, thyroid nodule diagnosis relies on both the properties of the nodule itself and its surrounding anatomical structures, such as the isthmus and adjacent lobes. Existing methods often struggle to accurately focus on key nodule regions when modeling global information. On the other hand, nodules exhibit significant morphological variations, including indistinct boundaries and irregular shapes, making it difficult for traditional CNNs to adapt to the complex variations of the nodules.

To address these issues, this paper proposes a multi‐scale feature fusion model that effectively integrates local nodule features with global contextual information, precisely focuses on the nodule region, and enhances the model's adaptability to nodule shape variations.

## 3 METHODS

### 3.1 Overview

**FIGURE 2:**
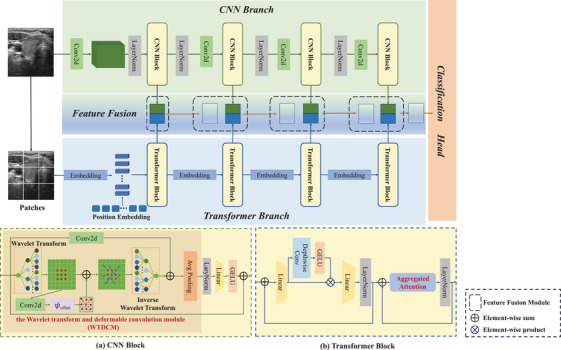
An overview of our proposed network for the classification of thyroid nodules. (a) and (b) are the detailed steps of the CNN block and transformer block. CNN, convolutional neural network.

The architecture of the proposed LGF‐Net model is illustrated in Figure 2. The architecture of this model consists mainly of three key elements: the CNN branch, the Transformer branch, and the feature fusion component. Using a parallel structure, multi‐scale local and global information from thyroid ultrasound images is extracted through the CNN and Transformer branches. The obtained features at each stage are then fused in the feature fusion component. Ultimately, a linear layer serves as a classification head to determine the benign or malignant nature of thyroid nodules. The following sections provide a comprehensive description of the proposed method.

### 3.2 WTDCM‐based CNN branch

To extract the texture features and fine‐grained information of thyroid nodules, we used the CNN branch to capture the local features of thyroid nodules. The CNN branch consists of four identical CNN blocks to extract local spatial information at different scales. The input ultrasound image $I\in R^{C\times H\times W}$ passes through convolutional downsampling and LayerNorm layers before entering the CNN blocks for feature learning, where C is the number of channels, H and W are the height and width of the image. Each time before entering the next CNN block, convolutional downsampling is applied, as shown in Figure 2.

Due to factors like tissue motion and morphological changes in the lesion area, the thyroid nodule region in ultrasound images may experience non‐rigid deformation. Traditional CNNs, relying on regular grid sampling methods, struggle to adapt to such complex spatial deformations. The deformable convolution network (DCN)^35^ introduces learnable offsets, allowing the convolutional kernel's sampling positions to adjust adaptively based on the image content. This enables the model to better handle spatial transformations in the image and focus on information‐dense areas. To generate these offsets dynamically, a lightweight offset prediction subnetwork is introduced within each CNN block. This subnetwork learns to output the offsets for each location on the feature map, effectively adjusting the sampling grid of the convolution kernels to better align with the underlying structure of the nodule region. By doing so, the model can capture deformed patterns more robustly and flexibly, especially in the presence of shape irregularities. Additionally, the wavelet transform enables multi‐scale decomposition of the image, capturing both low‐frequency global features and high‐frequency detailed features. To enhance the model's adaptability to deformations in thyroid nodule areas in ultrasound images and capture more effective features, we proposed a novel WTDCM, shown in Figure 2a. This structure leverages the advantages of both DCN and wavelet transform for convolution operations within the CNN blocks.

Specifically, the wavelet convolution is used to perform multi‐scale decomposition on the feature map $X\in R^{C\times H\times W}$, obtaining features in different frequency bands:

(1) $$\;x_{f}=\sum_{f\in \left\{LL,LH,HL,HH\right\}}conv\left(W_{f},X\right)\;,$$

where f represents the frequency band type, $W_{f}$ is the wavelet kernel, and LL,LH,HL,HH correspond to the low‐frequency and high‐frequency features obtained through wavelet transform in different directions.

To model spatial deformations, we predict learnable offsets based on the frequency‐decomposed features. Specifically, a 1×1 depthwise convolution layer followed by a linear layer is used to estimate the offset field:

(2) $$\Delta x=\psi_{offset}\left(x_{f}\right),$$

where $\psi_{offset}\left(\cdot \right)$ is the network that computes the offset, and $x_{f}$ represents the features from different frequency bands. These offsets are used to guide deformable convolutions on the corresponding frequency‐domain feature maps, as shown in Equation (3). By adjusting the sampling positions of the convolutional kernels, non‐uniform sampling on the image is achieved, enabling the model to flexibly and effectively focus on diagnostically critical regions even in the presence of spatial deformations.

(3)

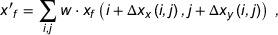

where $\Delta x_{x}\left(i,j\right)$) and $\Delta x_{y}\left(i,j\right)$ represent the offset values in the horizontal and vertical directions, and w is the weight of the convolution kernel.

Finally, a wavelet inverse convolution operation is performed to combine the features from each scale:

(4)

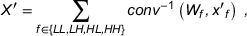




By combining the strengths of DCN and wavelet transform, this structure enhances the network's adaptability to complex deformations in ultrasound images and enables dynamic focusing on important regions at different scales, further improving the fine‐grained feature extraction capability.

### 3.3 Biologically‐inspired vision transformer branch

To mimic the biological visual information processing ability of doctors when diagnosing thyroid nodules and to capture global spatial information from thyroid ultrasound images, we introduced the AA mechanism^36^ in the design of the transformer branch. AA mechanism combines local sliding window attention and pooling attention to compute a refined attention matrix, facilitating the fusion of global and local information.

Specifically, the similarity matrices for the local sliding window and global pooling window are first calculated using the following equations:

(5) $$\;S_{\rho }=\left(\hat{Q}+\mathrm{QE}\right)K_{\rho }^{T},$$

(6) $$\;S_{\sigma }=\left(\hat{Q}+\mathrm{QE}\right)K_{\sigma }^{T},$$

where $K_{\rho }$ and $K_{\sigma }$ are the key matrices based on the local sliding window and global pooling window, $\hat{Q}$ represents the L2 normalized query matrix, and QE is a learnable query embedding to enhance the model's contextual awareness. The similarity matrices are then normalized using the Softmax function, yielding the final attention matrix:

(7) $$A\;\left(Q,K,V\right)=Softmax\left(\frac{\tau logN\times Concat\left(S_{\rho },S_{\sigma }\right)+B}{\sqrt{d_{k}}}\right),$$

where $Concat(S_{\rho },S_{\sigma })$ concatenates the local and global similarity matrices to incorporate both local and global information. logN and τ are parameters to adjust the scale and smoothness, $\sqrt{d_{k}}$ is a scaling factor, and B is the bias term.

To handle the influences of local and global attention separately, the attention matrix A(Q,K,V) is decomposed and weighted:

(8) $$A_{\rho },\;A_{\sigma }=\;\mathrm{Split}\left(A\right),$$

(9) $$AA\left(Q,K,V\right)=\left(A_{\rho }+{\hat{Q}}^{T}\right)V_{\rho }+A_{\sigma }V_{\sigma },$$

where $V_{\rho }$ and $V_{\sigma }$ are the value matrices corresponding to local and global attention, respectively, used to compute the final feature representation. Through this AA mechanism, the model can flexibly capture key information at different scales, ensuring sufficient perception of fine‐grained features around thyroid nodules while also attending to global information. This balances global and local information.

In our Transformer Branch, the input ultrasound image $I\in R^{C\times H\times W}$ is first divided into N=H/P×W/P patches, where P is the resolution of each image patch. After linear projection into the specified dimension space, the patches are combined with learnable position embeddings to generate patch embeddings. These embeddings then pass through four similar Transformer blocks, with linear projections applied before each block, as shown in Figure 2. In the final Transformer block, due to the small feature map size, pooling cannot be performed. Therefore, we replaced the AA mechanism with a multi‐head attention module^10^ to process the features at a smaller scale.

### 3.4 FFM‐enhanced feature fusion component

**FIGURE 3:**
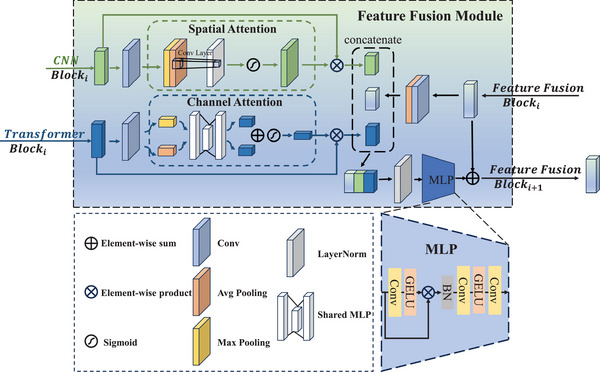
FFM. FFM, Feature Fusion Module.

The Transformer leverages the attention mechanism to capture long‐range dependencies, but it does not adequately strengthen the relationships between different channels. On the other hand, CNN generates local feature maps via sliding window operations. When dealing with relationships between distant pixels in an image, it overlooks the spatial dimension. To address the Transformer's limitations in the channel dimension and the spatial limitations of CNN, we adaptively combined local features from various layers, global representations, and synthesized semantic information, as shown in Figure 3.

Inspired by the use of channel attention (CA) and SA^30^ in feature processing, we applied a CA mechanism,^37^ using a multi‐layer perceptron (MLP) to weight the different channels of the global features extracted by the transformer block, thereby emphasizing important feature channels:

(10) $$\begin{array}{cc} X_{C} & =sigmoid\left(MLP\left(AvgPool\left(X_{TRANS}\right)\right)\right.\\ & \;\left.+MLP\left(MaxPool\left(X_{TRANS}\right)\right)\right), \end{array}$$

where $X_{C}$ denotes the feature extracted via the CA mechanism, and $X_{TRANS}\in R^{C\times H\times W}$ represents the feature extracted by the Transformer Block.

The SA mechanism^38^ strengthens CNN's ability to focus on key spatial regions within the feature map $X_{CNN}\in R^{C\times H\times W}$, further enhancing the model's capacity for local feature representation:

(11) $$\begin{array}{cc} X_{S} & =sigmoid\\ & \times \left(f^{7\times 7}Concat\left(MaxPool\left(X_{CNN}\right),AvePool\left(X_{CNN}\right)\right)\right), \end{array}$$

where $X_{S}$ is the feature obtained through the SA mechanism, $X_{CNN}\in R^{C\times H\times W}$ is the feature extracted by the CNN Block, and $f^{7\times 7}$ represents a 7×7 convolution kernel.

The feature fusion component, which includes four individual FFMs, integrates both global and local features that originate from the outputs of four distinct stages. In the first FFM, the local and global features are fused through an MLP and then passed into the next FFM. In subsequent fusion modules, the features from the previous layer, as well as the global and local features from the current layer, are merged, as shown in Figure 3. The fusion process can be expressed as:

(12) XFi=MLPLNXSi∗XCNNi,XCi∗XTRANSi,AvgPool(XFi−1)Concat×XSi∗XCNNi,XCi∗XTRANSi,AvgPool(XFi−1)+XFi−1,

where $X_{F}^{i}$ represents the fused features at the i‐th stage, and LN denotes the LayerNorm operation.

## 4 EXPERIMENT SETTINGS

### 4.1 Dataset

The effectiveness of LGF‐Net is validated using two datasets. One of them is the thyroid nodule classification dataset (TNCD),^39^ which is tailored for thyroid nodule classification tasks. This dataset includes 3,493 ultrasound images collected from 2,421 patients, with each image classified as benign or malignant based on cytological biopsy results. To assess the algorithm's performance, the TNCD dataset is divided into a training set and a test set, ensuring that images from the same patient appear in only one of the subsets. The training set comprises 2879 images, consisting of 1905 benign and 974 malignant images, while the test set includes 614 images, with 378 benign and 236 malignant images. The second dataset is a local dataset collected from West China Hospital in Chengdu, China, consisting of 3534 two‐dimensional (2D) B‐mode ultrasound images of thyroid nodules, including 2408 benign and 1126 malignant nodule images. All images were acquired by professional ultrasound physicians, and the nodule labels were determined based on fine‐needle aspiration biopsy (FNAB) biopsy reports. The dataset was anonymized after obtaining patient consent to ensure privacy protection, and the training and test sets were split at an 8:2 ratio. Our dataset is highly representative, encompassing both transverse and longitudinal images. Moreover, it captures the diversity of thyroid nodules in terms of echogenicity, composition, margins, and location, making it well‐suited for clinical classification research. The evaluation on these two datasets was conducted independently.

### 4.2 Evaluation metrics

To quantitatively assess the performance of the proposed method, we used four evaluation metrics: Accuracy, Recall, Precision, and F1‐score, all of which are calculated based on the confusion matrix. These metrics are defined as follows:

(13) $$Accuracy=\frac{TP+TN}{TP+FP+TN+FN}\;,$$

(14) $$Recall=\frac{TP}{TP+FN}\;,$$

(15) $$Precision=\frac{TP}{TP+FP}\;,$$

(16) $$F1-\mathrm{score}\;=2\times \;\frac{Recall\times Precision}{Recall+Precision}=\frac{2TP}{2TP+FP+FN}\;,$$

where TP, TN, FP, and FN are defined by True Positive, True Negative, False Positive, and False Negative.

### 4.3 Implementation details

All models were trained on a system equipped with an NVIDIA GeForce RTX 3060 (12 GB memory) running CUDA 12.6. The programming environment used Python 3.7.13, and the implementation was based on PyTorch 1.8.1+cu111. For the CNN branch, we initialized all layers using random values, while for the Transformer branch, we initialized the attention layer and other Transformer‐related components using ImageNet pre‐trained weights. The remaining layers were initialized randomly. During training, the images were resized to 224×224 and augmented through random flipping, cropping, and color jittering, all of which were implemented using PyTorch's library. All images were normalized using the mean [0.485, 0.456, 0.406] and standard deviation [0.229, 0.224, 0.225] from the ImageNet dataset. In the experiments, we used Label Smoothing Cross‐Entropy Loss to decrease the loss weight for samples that are easily classified, which helps the model focus more on challenging examples and reduces the risk of overfitting to the training data, as shown in Equation (17):

(17) $$\begin{array}{cc} & Label\;Smoothing\;Cross-Entropy\;Loss\\ & \;=-\sum_{i}^{N}\sum_{j=1}^{C}\left(\left(1-\varepsilon \right)y_{ij}+\frac{\varepsilon }{C}\right)\log\left(p_{ij}\right), \end{array}$$

where ε is the smoothing parameter ranging from [0, 1], used to smooth the ground truth labels. N denotes the number of samples, and C represents the number of classes. $y_{ij}$ denotes the ground truth label for class i, and $p_{ij}$ is the predicted probability that the i‐th sample belongs to class j. Detailed parameter settings are provided in Table 1.

**TABLE 1:** Experimental setting.

Training config	Setting
Training epochs	300
Batch size	32
Optimizer	AdamW^40^
Optimizer momentum	𝛽1, 𝛽2 = 0.9, 0.999
Drop path rate	0.7
Base learning rate	5e−4
Min learning rate	1e−6
Learning rate schedule	CosineAnnealingLR
Weight decay	0.05
Warm up schedule	linear
Warm up epochs	5

## 5 RESULTS

### 5.1 Performance comparison with state‐of‐the‐art methods

To comprehensively evaluate the performance of our LGF‐Net, we compared it with state‐of‐the‐art methods from recent years, categorized into CNN‐based methods, Transformer‐based methods, and CNN‐Transformer hybrid methods. Specifically, the CNN‐based methods include ResNet50,^41^ Mobilenet‐v3,^42^ and ConvNeXt.^43^ The Transformer‐based methods include VIT,^11^ Swin‐v2,^44^ and TransNext.^36^ The CNN‐Transformer hybrid methods include Mobilevit,^45^ MobileFormer,^46^ UniFormer,^47^ Conformer,^48^ MedViT,^29^ and Hifuse.^30^ Additionally, we selected several networks specifically designed for thyroid nodule classification, such as ACL‐Resnet,^39^ LoGo‐Net,^25^ SDA‐Net101^24^, Hybrid Google,^49^ and IFormer‐DVNet.^50^ These methods were implemented and applied to the dataset used in this study, with the main architecture and hyperparameters consistent with those reported in their respective papers, as shown in Table 1.

In Table 2, it is evident that the proposed LGF‐Net outperforms previous methods in all four metrics—Accuracy, Recall, Precision, and F1‐Score—on the TNCD dataset, achieving an average Accuracy of 81.50%, Recall of 79.51%, Precision of 79.92%, and F1‐Score of 79.70%. Similarly, in Table 3, LGF‐Net demonstrates superior overall performance on the local dataset compared to previous methods. Experimental results show that LGF‐Net effectively performs accurate thyroid ultrasound nodule classification and addresses the irregular variations in nodules by learning both local and global features. Additionally, our local dataset, collected from actual clinical diagnoses, further validates LGF‐Net's effectiveness in real‐world clinical environments and across domain datasets.

**TABLE 2:** Comparisons with the state‐of‐the‐art semantic classification models on the TNCD dataset.

Models	Accuracy (%)	Recall (%)	Precision (%)	F1‐score (%)
ResNet50^41^	68.59	62.63	68.37	62.38
Mobilenet‐v3^42^	74.34	71.13	73.50	71.76
VIT‐B‐16^11^	71.22	67.49	70.00	67.99
Swin‐v2‐B^44^	74.18	72.08	72.85	72.38
ConvNeXt‐B^43^	76.64	74.79	75.50	75.09
TransNext‐B^36^	76.87	74.61	75.76	75.05
Mobilevit‐S^45^	63.32	54.23	63.06	49.15
MobileFormer‐52m^46^	64.36	63.91	62.93	63.30
UniFormer‐B^47^	72.86	69.46	71.83	70.03
Conformer‐B‐16^48^	77.04	76.10	75.75	75.91
MedViT‐B^29^	75.73	72.49	74.98	73.19
Hifuse‐B^30^	73.78	72.44	70.98	71.46
ACL‐Resnet^39^	73.28	73.64	63.02	67.87
LoGo‐Net^25^	62.05	57.16	58.56	56.97
SDA‐Net101^24^	75.57	73.55	74.24	73.84
Hybrid Google^49^	77.20	77.97	67.65	72.44
IFormer‐DVNet^50^	76.55	76.55	76.36	75.85
Ours	**81.50**	**79.51**	**79.92**	**79.70**

*Note*: the best results are highlighted in bold, while the second‐best results are underscored.

Abbreviation: TNCD, thyroid nodule classification dataset.

**TABLE 3:** Comparisons with the state‐of‐the‐art semantic classification models on the local dataset.

Models	Accuracy (%)	Recall (%)	Precision (%)	F1‐score (%)
ResNet50^41^	84.23	79.46	83.18	80.87
Mobilenet‐v3^42^	85.51	83.23	83.38	83.30
VIT‐B‐16^11^	83.52	79.41	81.73	80.37
Swin‐v2‐B^44^	87.22	83.89	86.13	84.85
ConvNeXt‐B^43^	88.21	85.80	86.79	86.26
TransNext‐B^36^	90.51	88.66	89.33	88.98
Mobilevit‐S^45^	77.98	72.51	75.01	73.45
MobileFormer‐52m^46^	66.52	64.29	64.21	64.25
UniFormer‐B^47^	86.79	82.87	86.04	84.16
Conformer‐B‐16^48^	90.65	**89.35**	89.17	89.26
MedViT‐B^29^	90.51	88.07	89.77	88.84
Hifuse‐B^30^	89.94	86.94	89.51	88.05
ACL‐Resnet^39^	87.11	84.04	85.76	84.81
SDA‐Net101^24^	90.23	88.92	88.65	88.79
Ours	**91.24**	88.90	**90.73**	**89.73**

*Note*: the best results are highlighted in bold, while the second‐best results are underscored.

The standalone CNN‐based or Transformer‐based baseline models, such as ResNet50, Mobilenetv3, and VIT, performed poorly, likely due to these models being primarily designed for natural images. Thanks to the AA mechanism, TransNext achieved the best results among these baseline models. Conformer also ranked highly on both datasets, showcasing the advantage of multi‐branch fusion between CNN and Transformer. In contrast, our method eliminates the need for information exchange between the CNN and Transformer branches, opting instead to merge features from different blocks. This approach reduces computational complexity while enhancing classification accuracy. Models specifically designed for medical images, such as MedViT and Hifuse, as well as thyroid nodule‐specific models like ACL‐Resnet, LoGo‐Net, and SDA‐Net, showed unstable performance across different datasets, being more susceptible to domain shifts. The hybrid Google and IFormer‐DVNet did not provide open‐source code, making them unavailable for experimentation on the local dataset, and thus are not included in the comparison.

**FIGURE 4:**
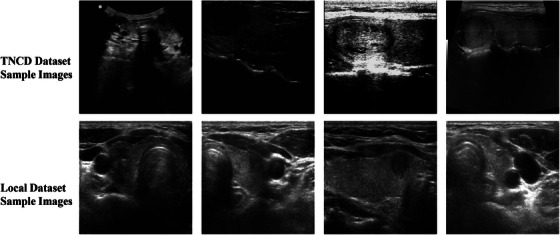
Comparison between the TNCD dataset and the local dataset. TNCD, thyroid nodule classification dataset.

Furthermore, we observed that all models performed better on the local dataset than on the TNCD dataset across the four metrics, which may be attributed to the higher quality and resolution of the thyroid ultrasound images in the local dataset, as shown in Figure 4. Compared to the TNCD dataset, the images in the local dataset are closer to real‐world clinical environments, providing clearer features of nodules and anatomical structures. This helps the model better identify the thyroid nodule regions and extract more precise features, leading to improved classification performance on the local dataset.

Compared to previous methods, LGF‐Net not only captures the local features of the nodules but also attends to the global contextual features, guiding the model to learn more accurate classification results. This enhances classification performance and demonstrates stable results across datasets from different sources.

**FIGURE 5:**
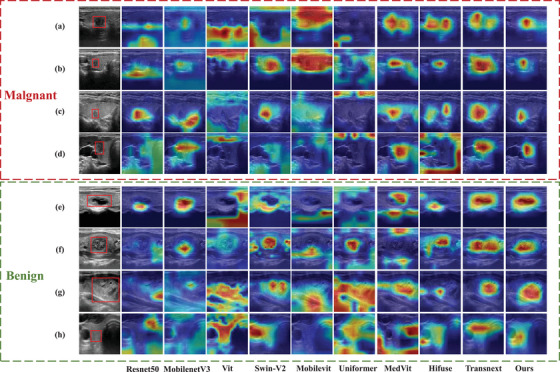
The visualization of Grad‐CAM in different models. The red boxes indicate the nodules. (a) and (b) are malignant nodules from the TNCD dataset, (c) and (d) are malignant nodules from the local dataset, (e) and (f) are benign nodules from the TNCD dataset, and (g) and (h) are benign nodules from the local dataset. TNCD, thyroid nodule classification dataset.

To further demonstrate that the proposed method accurately captures nodule features, we visualized the results of LGF‐Net and previous methods using Grad‐CAM,^51^ as shown in Figure 5. In the figure, the first column on the left shows the original ultrasound images, with the red boxes indicating the thyroid nodule regions. It can be observed that ResNet50, ViT, Mobilevit, and UniFormer are easily influenced by background information, focusing more on irrelevant areas rather than the nodule region. MobilenetV3, Swin‐V2, MedViT, and Hifuse are generally able to locate the nodule region, but their localization is imprecise. Notably, except for LGF‐Net, TransNext is also able to accurately perceive the nodule location, likely due to the attention mechanism used in TransNext, which helps focus on important regions. This also highlights the ability of LGF‐Net to capture nodule features precisely. Furthermore, for the benign nodule shown in Figure 5h, which is less obvious, only LGF‐Net effectively focuses on the corresponding region. These results confirm the model's ability to focus on relevant features and indicate that attention to important features can significantly improve model performance.

### 5.2 Ablation study

To evaluate the impact of each component on the model, we conducted ablation experiments on the TNCD dataset, analyzing the CNN Branch, CNN Branch with WTDCM, Transformer Branch, Transformer Branch with AA mechanism, and the Fusion Module, as shown in the “Component” section of Table 4. When using only the CNN Branch or the Transformer Branch, the model's performance was suboptimal, with accuracies of 72.93% and 73.08%, respectively. However, after incorporating the WTDCM and AA mechanism, all four metrics showed improvements, indicating that the WTDCM and AA mechanism play a positive role in enhancing the network's performance. Furthermore, the FFM efficiently integrates both local and global features from the two branches, resulting in an accuracy of 81.50%. Furthermore, we evaluated the performance of different stage fusion configurations of the two branches on the TNCD dataset. As shown in the “Stage” section of Table 4, applying the Fusion Module at more stages yields better results, with the optimal performance achieved when fusion occurs at all four stages. This ablation study further validates the necessity of performing fusion at multiple stages, as it enables a more comprehensive integration of both global and local features.

**TABLE 4:** Ablation experiment results on the TNCD dataset.

	CNN Branch	WTDCM	Transformer Branch	AA Mechanism	FFM	Accuracy (%)	Recall (%)	Precision (%)	F1‐score (%)
**Component**	√	×	×	×	×	72.93	68.14	70.59	68.84
	√	√	×	×	×	76.99	72.81	75.47	73.68
	×	×	√	×	×	73.08	68.45	70.74	69.13
	×	×	√	√	×	77.52	74.50	76.98	75.25
	√	√	√	√	√	81.50	79.51	79.92	79.70

		Stage 1	Stage 2	Stage 3	Stage 4	Accuracy (%)	Recall (%)	Precision (%)	F1‐score (%)
**Stage**		×	×	×	√	76.24	70.43	75.76	71.59
		×	×	√	√	77.29	72.66	76.15	73.69
		×	√	√	√	79.15	75.03	80.49	76.21
		√	√	√	√	81.50	79.51	79.92	79.70

Abbreviations: AA, aggregated attention; FFM, feature fusion module; TNCD, thyroid nodule classification dataset; WTDCM, wavelet transform and deformable convolution module.

## 6 DISCUSSION

In the task of thyroid nodule detection, capturing multi‐scale global‐local features from ultrasound images demonstrates significant advantages. Moreover, due to the subtle differences and irregular deformations of nodules, accurately focusing on important regions and adapting to nodule variations are also challenges that need to be addressed. Our proposed method processes the features of thyroid nodules in three distinct ways: First, instead of relying on standard convolutions to extract local features like nodule textures, we introduced WTDCM, which incorporates deformable convolutions with additional offsets to handle the irregular deformations of nodules. We also used wavelet transforms for multi‐scale image decomposition, capturing both low‐frequency and high‐frequency features, which improves the precision of feature extraction. Second, we employed a transformer branch combined with the AA mechanism to locate the nodule regions precisely and extract richer contextual information, considering factors such as position and background. Finally, the features from different branches were integrated by FFM, with spatial and CA utilized to enhance relevant features and suppress irrelevant ones, thus improving branch performance.

**FIGURE 6:**
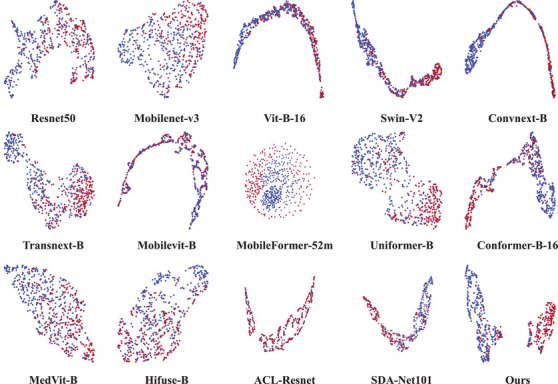
Visualization of learned features using UMAP for different methods. The blue and red points represent benign and malignant nodules. UMAP, uniform manifold approximation and projection.

To further evaluate our model's effectiveness, we applied the uniform manifold approximation and projection (UMAP) algorithm to perform dimensionality reduction on the deep features learned by various models from the TNCD dataset and visualized the outcomes in Figure 6. Although most methods demonstrate relatively compact intra‐class clusters, they also exhibit a high degree of overlap between classes. This suggests that while these methods effectively classify nodules with distinct features, they struggle to differentiate nodules with subtle differences. In contrast, LGF‐Net achieves larger inter‐class separation while maintaining more compact intra‐class clustering, indicating its superior classification performance and enhanced discriminative ability.

As demonstrated in Section 5, LGF‐Net consistently achieves outstanding classification performance across multiple thyroid nodule datasets. The visualization results further confirm that LGF‐Net effectively focuses on nodule regions, reinforcing the model's interpretability and potential clinical significance. Additionally, we found that image quality is a non‐negligible factor affecting the results. Although the ultrasound images used in our study come from multiple datasets, their quality and resolution vary. In particular, some images in the TNCD dataset have lower quality, which may have impacted the model's performance on this dataset. Lower image quality typically leads to noise and blurring, increasing the difficulty of feature extraction for the model and thus affecting classification results. In contrast, the images in the local dataset were all collected under clinical guidelines, providing clearer nodule features, which allowed the model to more accurately extract features and make more precise classification decisions.

Although the method demonstrates excellent performance in nodule diagnosis, there are still some limitations. On one hand, the dataset used to build and evaluate the model consists of single‐modality grayscale ultrasound images, with classification performed based on individual frames. However, in actual clinical diagnoses, thyroid data are often presented as video streams, and doctors may also refer to multimodal data, such as Doppler ultrasound, computed tomography (CT), and magnetic resonance imaging (MRI). Relying solely on single‐frame grayscale ultrasound images limits the model's ability to leverage different types of information. On the other hand, the ultrasound images in our dataset are labeled as either benign or malignant, with no additional information. More precise annotations of nodule features, such as shape, boundaries, calcification, etc., could potentially improve diagnostic performance. Therefore, in future work, we aim to enhance diagnostic performance by refining nodule features and incorporating both temporal information from video streams and spatial data from single‐frame images. Also, we plan to leverage other modalities, such as Doppler ultrasound for blood flow data and CT images to capture the relationship between lesions and surrounding tissues. Additionally, we will explore ways to improve the model's performance on low‐quality images, further enhancing its adaptability in various clinical environments.

## 7 CONCLUSIONS

The proposed LGF‐Net significantly improves thyroid nodule classification performance in ultrasound images by fusing local and global features at multiple scales. In the CNN branch, the WTDCM, which combines wavelet convolutions and deformable convolutions, effectively adapts to the non‐rigid deformations of nodules and extracts multi‐scale local features. In the Transformer branch, the AA mechanism helps the model focus on the nodule region and capture global information. The FFM is designed to efficiently integrate global and local features while blocking information exchange between the two branches, ensuring the independence of global and local feature extraction and preventing the introduction of additional noise. Experimental results show that the proposed method significantly improves thyroid nodule classification performance and enhances the model's interpretability by providing the location of diagnostic features. Experiments on multi‐center datasets validate the robustness and stability of LGF‐Net, providing important insights for optimizing clinical diagnosis and improving diagnostic effectiveness.

## AUTHOR CONTRIBUTIONS

**Yao Xiao**: Conceptualization; methodology; software; formal analysis; investigation; data curation; writing—original draft preparation; writing review and editing; visualization. **Yan Zhuang**: Conceptualization; methodology; formal analysis; writing—review and editing; supervision. **Wenwu Ling**: Formal analysis; resources. **Shouyu Jiang**: Software; data curation. **Ke Chen**: Software. **Guoliang Liao**: Data curation. **Yuhua Xie**: Software. **Yao Hou**: Data curation. **Lin Han**: Software. **Zhan Hua**: Conceptualization; supervision. **Yan Luo**: Resources. **Jiangli Lin**: Conceptualization; methodology; formal analysis; writing review and editing, supervision. Jiangli Lin and Zhan Hua All authors have read and agreed to the published version of the manuscript.

## CONFLICT OF INTEREST STATEMENT

The authors declare no conflicts of interest.

## ETHICS STATEMENT

The study was conducted in accordance with the Declaration of Helsinki and approved by the Ethics Committee of West China Hospital of Sichuan University Biomedical Research. Informed consent was obtained from all subjects involved in the study.

## Data Availability

Authors will share data upon request to the corresponding author.
